# Comparison of the effects of reconstruction of the lateral ankle ligaments using peroneus longus and peroneus brevis tendon graft

**DOI:** 10.1097/MD.0000000000022912

**Published:** 2020-11-13

**Authors:** Zongyu Yang, Fei Liu, Liang Cui, Heda Liu, Junshui Zuo, Lin Liu, Sentian Li

**Affiliations:** Department of Sports Medicine - Foot and Ankle Surgery, Cangzhou Hospital of Integrated TCM-WM, Cangzhou, Hebei, PR China.

**Keywords:** chronic lateral ankle instability, lateral ankle ligament reconstruction, peroneus brevis tendon., peroneus longus tendon

## Abstract

Peroneus longus and peroneus brevis tendon grafts have been frequently used to reconstruct the lateral ankle ligaments. However, there is no literature comparing the effect of the 2 methods. The purpose of this study was to compare the effects of 2 autologous tendon transplants on ankle joint activity.

This retrospective study included 100 adult patients with chronic lateral ankle instability (CLAI) who underwent surgery from January 2014 to December 2017. Group A (50 patients): Reconstruction of the lateral ankle ligaments using the anterior half of peroneus longus tendon graft; Group B (50 patients): Using the anterior half of peroneus brevis tendon graft. Outcomes were assessed by comparing pre- and postoperative AOFAS scores, VAS pain scores, and Karlsson scores, and the radiographic assessment included talar tilt and anterior talar translation. A sensitive dynamometer was used before and after surgery to assess inversion, valgus, plantarflexion, and dorsiflexion strength to evaluate changes in muscle strength in the patients feet.

Postoperatively, 88 patients were followed up for 12 to 24 months, including 46 cases in group A and 42 in group B. No severe complications were recorded in the 2 groups. There were significant pre- to post-operative differences between the groups. No significant differences were observed in the postoperative scores and muscle strength changes between the groups. However, the number of patients with decreased valgus strength in group B was statistically significant compared with group A.

Both methods can improve the stability of the ankle joint, but the peroneus longus tendon has little effect on the postoperative muscle strength of the foot and should be used as the preferred surgical treatment for the treatment of CLAI.

## 1 Introduction

When conservative treatment fails for chronic lateral ankle instability (CLAI), the main surgical treatment is the Broström-Gould procedure, or autograft and allograft tendon reconstruction. The Broström-Gould method is not applicable to patients with anterior talofibular ligament (ATFL) and calcaneofibular ligament (CFL) residual deficiencies, systemic ligament relaxation, and obesity.^[1]^ The potential complications of allograft reconstruction of the lateral collateral ligament of the ankle include disease and infection transmission, subclinical immune responses, and increased cost.^[2,3]^ In recent years, the use of autologous tendon in the treatment of CLAI has become a relatively accepted surgical treatment.

Presently, the reconstruction of the lateral ankle ligament with the anterior half of the peroneus longus tendon (AHPLT) and the anterior half of the peroneus brevis tendon (AHPBT) is a common treatment method, and satisfactory results have been achieved without surgical complications. As early as 1999, Sammarco et al reported that 30 patients with chronic lateral ankle instability were reconstructed using a split peroneus brevis tendon graft. After an average of 44 months of follow-up, this procedure was reliable with a 94% rate of good to excellent clinical results and a 97% rate of mechanical stability.^[4]^ In 2012, Zhao et al^[5]^ found that cutting the peroneus longus tendon had no effect on ankle joint stability. Kim et al^[6]^ reported that use of the AHPLT to reconstruct the lateral ligament achieved good results. Sun et al studied 32 consecutive patients with CLAI who underwent AHPLT transfer, and found that postoperative AOFAS scores, talar tilt, and anterior talar translation were greatly improved.^[7]^

Although many reports have described satisfactory results, tendons have unique functions, and the normal tissue is sacrificed after reconstruction of the autologous tendon, which may change foot biomechanics and limit joint activity.^[8,9]^ To our knowledge, the effects of AHPLT and AHPBT reconstruction on foot activity (inversion, valgus, plantarflexion, and dorsiflexion) have not been compared in the literature. The purpose of this study was to determine which tendon has less impact on the foot, is safer, and is more suitable for ligament reconstruction.

## 2 Methods

This retrospective study included 100 adult patients with chronic lateral ankle instability (CLAI) who underwent surgery from January 2014 to December 2017. The protocol was approved by our local ethics committee. All patients provided informed written consent prior to treatment and study participation. Patients with a primary diagnosis of CLAI who satisfied the inclusion and exclusion criteria (Table 1) were recruited from the outpatient orthopedics department.

**Table 1:** Inclusion and exclusion criteria.

Inclusion Criteria
Patients diagnosed with CLAI
Failure of conservative treatment more than 6months
Ankle pain and swelling
giving way sensation
evidence of ankle instability on clinical or radiography examination (positive anterior drawer and talus tilt
Exclusion Criteria
Any history of surgery treatment for ankle
Medial (deltoid ligament) instability
Local infection of the foot
Deformity of foot and ankle (flatfoot, clubfoot, tarsal coalition, etc.)
Body weight over 120Kg
Severe medical department diseases (lesions affecting liver and kidney function, severe diabetes and heart disease, central nervous system diseases)

Group A (50 patients): Reconstruction of the lateral ankle ligaments using the anterior half of peroneus longus tendon graft; Group B (50 patients): Using the anterior half of peroneus brevis tendon graft. The mean age of patients in the AHPLT group (39.9 ± 9.26 years) and in the AHPBT group (35.56 ± 11.8 years) was comparable (*P* = .651). There were an equal number of male and female patients in the AHPLT group (n = 25 each), whereas the number of males (n = 26) was higher than females (n = 24) in the AHPBT group (*P* = .19). Average follow up time in the 2 groups was 22 (range, 12–36) months.

### 2.1 Clinical outcome assessment and data collection

Pre- and post-operatively during the follow-up period, the American Orthopedic Foot & Ankle Society (AOFAS) Ankle-Hindfoot Scale^[10]^ and the Visual Analog Scale (VAS) were used to determine clinical outcomes and associated levels of pain.

The AOFAS score mainly includes ankle pain, function, and force line evaluation and has a maximum score of 100. An ankle evaluation of 90 to 100 points indicates that the outcome is excellent, 80 to 89 points is a good outcome, 70 to 79 is a fair outcome, and 70 points or less means poor recovery after surgery. The VAS score ranges from 0 to 10 points, with no pain being 0 points, mild pain being 1 to 3 points, moderate pain being 4 to 6 points, and severe pain being 7 to 10 points. The Karlsson-Peterson Score^[11]^ was calculated at the last follow-up. (Table 2)

**Table 2:** Clinical rating scale for postoperative ankle reconstruction^[10]^.

Rating	Description
Excellent	Full range of motion equal to the contralateral ankle without pain. Able to return to the preinjury level and unrestricted work or sports activity
Good	Functional range of motion and stable ankle. Able to return to the preinjury level with minimal pain with work or sport activity
Fair	Functional range of motion, good stability, moderate level of pain, and/or stiffness with activities of daily living and sports activity.
Poor	Persistent instability or pain, the same or worse than before surgery.

**Figure 1:**
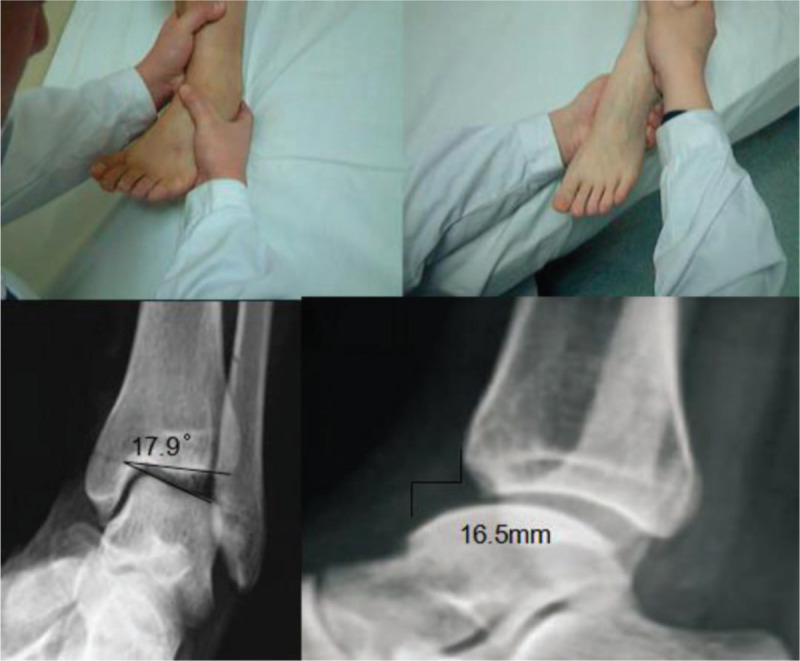
Talus varus stress test and anterior drawer test. Preoperative stress radiographs of a patient: the talar tilt angle was 17.9°; the anterior talar translation was 16.5 mm.

Radiologic examinations were performed, including the talar tilt test and the anterior drawer test. The talar tilt and anterior talar translation distance were recorded during the patients preoperative assessment and final follow-up. Under an X-ray in the stressed position, a talar tilt angle greater than 9° or a difference of more than 3° between the feet indicates that the talar varus test is positive; when the talus moves forward by more than 10 mm or exceeds the contralateral side by 3 mm, the anterior drawer test is positive^[12,13]^ (Fig. 1).

The patient was in the supine position and the ankle was in the neutral position. The sensitive dynamometer was used to measure the bilateral inversion, valgus, plantarflexion, and dorsiflexion extension strength. The dynamometer readings were recorded in detail to determine the muscle strength of the patient at the final follow up. To make comparisons with preoperative values and those of the healthy side, and in order to reduce error during the measurement, 2 physicians measured the muscle strength in the same patient, and each physician performed 5 measurements that were then averaged.

### 2.2 Operative techniques

All patients were operated on by the same chief physician. The patient was placed in the supine position, and after epidural anesthesia, the assistant surgeon wore a lead apron and performed the anterior talus test and talus varus test under X-ray to confirm instability of the ankle joint, which was helpful for the postoperative comparison. A 30-degree arthroscope with a 2.7-mm diameter was used. Anterior-medial and anterior-lateral approaches to the ankle were performed. First, the degree of joint capsule and ligament injury was assessed. The synovium that had proliferated in the ankle joint cavity was removed and the arthroscope was withdrawn. A curvilinear incision was then made approximately 7 cm below the lateral malleolus and along the peroneal muscle, the sural nerve was protected, the injured ligaments were exposed, and the avulsed fragments at the tip of lateral malleolus were removed. The peroneus longus (brevis) tendon was exposed, and the peroneus longus (brevis) tendon was split longitudinally at the transitional point of the tendon abdomen and the anterior bundle was cut. The distal fibula was drilled with a 5-mm drill through the fibula. The bone tunnels were drilled with a diameter of 0.6 cm at the calcaneofibular and anterior talofibular ligaments. In a neutral ankle position with slight external rotation, 1 end of the peroneus longus (brevis) tendon was fixed at the end of the anterior talofibular ligament with an interfacial screw. The tendon was passed through the fibular tunnel to tighten the tendon, fixed to the other end of the tendon in the calcaneal tunnel, and the sutured was strengthened with the surrounding tissue. The wound was then washed and sutured closed.

Postoperative, the ankle was splinted with plaster at 90 degrees. The contraction training of the quadriceps and hip muscles began on the hospital bed the next day after surgery. On the third day, holding crutches, partial weight-bearing. After 2 weeks, the wound suture was removed; after 4 weeks, the ankle inversion, valgus, plantarflexion, and dorsiflexion were gradually exercised. Anti-resistance training for ankle joint inversion, valgus, plantarflexion, and dorsiflexion was performed 6 weeks after surgery. Eight weeks after surgery, the daily activities were gradually restored.

### 2.3 Efficacy evaluation criteria

At the last follow-up, subjective patient satisfaction was recorded. We evaluated whether the ankle joint returned to a stable state, whether the range of motion was normal compared with the contralateral ankle joint, and whether the ankle was painful during normal work or activity. In addition, the patients were asked if they had pain or swelling on the other side of the ankle, and if they were willing to undergo ligament reconstruction surgery again or whether surgery was recommended for patients with the same disease.

Outcomes were assessed by comparing the pre- and post-operative American Orthopedic Foot and Ankle Society (AOFAS) scores, Visual Analog Scale (VAS) pain scores, and Karlsson scores. All patients underwent pre- and post-operative radiographic assessment including talar tilt and anterior talar translation. Simultaneously, we assessed muscle strength changes in the feet.

### 2.4 Statistical analysis

The statistical analysis was performed using SPSS Statistics version 16.0 software (SPSS Inc., Chicago, Illinois, USA). A paired *t* test was used to compare pre- and post-operative values (AOFAS ankle-hindfoot scores, VAS scores, Karlsson ankle scores, and radiologic measurement, and patients’ inversion, valgus, plantarflexion, and dorsiflexion strength). Statistical significance was accepted for *P* values <.05.

## 3 Results

**Figure 2:**
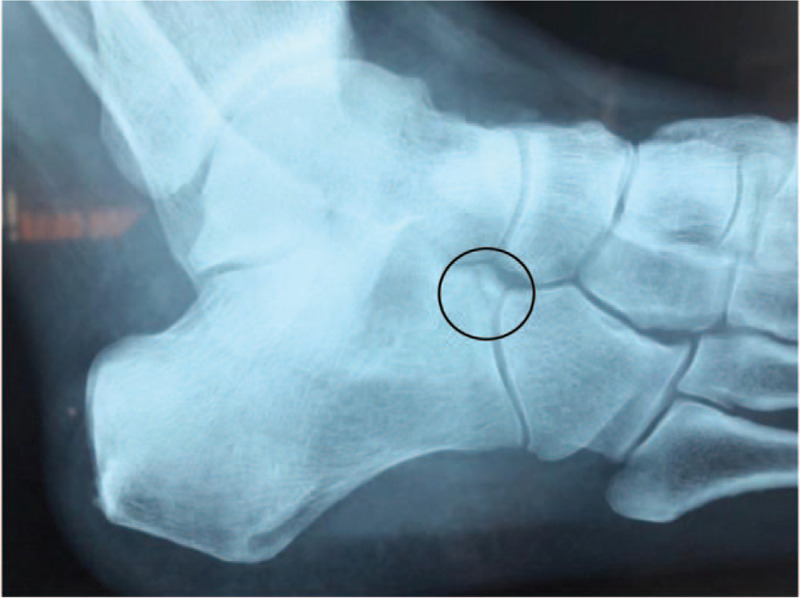
Surgical procedure for reconstructing the lateral ligament of the ankle with the peroneus brevis tendon.

**Figure 3:**
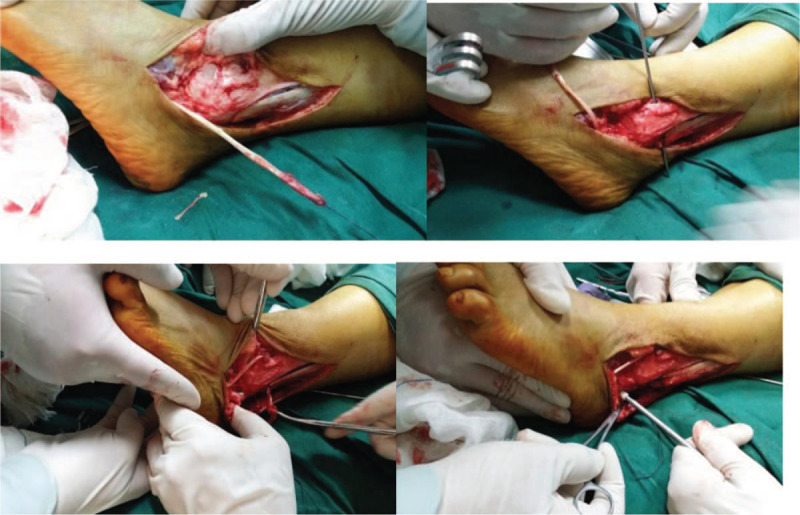
Avulsion fracture of calcaneal anterior tubercle.

Postoperatively, 88 patients (88 ankles) (88%) returned for a final evaluation, including 46 patients in group A and 42 in group B. In group A (AHPLT), overall patient satisfaction was rated as excellent by 40 (87.0%) patients, good by 2 (4.3%) patients, fair by 2 (4.3%) patients, and poor by 2 (4.3%) patients. One patient who was dissatisfied with the results complained of swelling and pain at the calcaneocuboid joint 1 year after surgery. A detailed examination of this patients imaging data (in particular, a preoperative oblique X-ray of the foot (Fig. 2) before and after surgery showed that the patient had a small avulsed bone in the anterior calcaneus nodules, which was not treated at that time. The other patient complained of a mass and tenderness at the incision at the final follow up, so the patient received an ankle MRI, which was unremarkable (Fig. 3).

In group B (AHPBT), 39 patients rated their outcome as good to excellent. No patient rated their outcome as fair, and 3 patients had a poor outcome. One patients outcome was highly unfortunate. After 1 year, he suffered a car accident that led to amputation of both of his lower extremities. There was also a male patient who had severe pain in the first metatarsophalangeal joint 4 months after surgery and was unable to move. Uric acid levels were found to be 762 μmol/L, and the diagnosis of gout was made. Two years after surgery, a large number of tophi was found in the patients foot. The last patient was dissatisfied because the patient had a scar and was not satisfied with the postoperative scar. However, the patient had achieved mechanical stability on ankle stress radiography. No statistically significant difference was observed compared with group A (Table 3).

**Table 3:** Subjective satisfaction score in 2 Groups.

	Excellent	Good	Fair	Poor
A (n = 46)	40 (87.0%)	2 (4.3%)	2 (4.3%)	2 (4.3%)
B (n = 42)	35 (83.3%)	4 (9.5%)	0 (0%)	3 (7.1%)

Preoperatively, the mean AOFAS scores in group A (56.7 ± 7.2) and group B (57.9 ± 6.4) were comparable (*P* = .672). Postoperatively, the score improved considerably in each group at each follow-up. The mean preoperative VAS score was 6.8 ± 0.9 in group A and 6.4 ± 1.0 in group B, and this difference was not significant (*P* = .216). Postoperatively, there was a downward trend in the values of the VAS score in both groups at the final follow up. However, with the numbers available, the difference in the AOFAS and VAS scores between the groups (group A and group B) was not statistically significant (Table 4).

**Table 4:** Preoperative and final follow-up values of the assessed variables.

	Preoperative	Last follow-up	*P* value	Test statistic
AOFAS (points)				
Group A	56.7 ± 7.2	91.3 ± 5.1	*P* < .05	−23.131
Group B	57.9 ± 6.4	90.7 ± 6.2	*P* < .05	−19.927
VAS (points)				
Group A	6.8 ± 0.9	1.4 ± 0.5	*P* < .05	8.256
Group B	6.4 ± 1.0	1.1 ± 0.9	*P* < .05	7.115
Karlsson (points)				
Group A	60.2 ± 5.7	89.2 ± 4.4	*P* < .05	−19.185
Group B	58.4 ± 6.9	86.4 ± 6.0	*P* < .05	−20.375
Anterior displacement (mm‘ x ± s)				
Group A	14.8 ± 3.4	2.9 ± 1.7	*P* < .05	14.859
Group B	15.0 ± 2.5	3.0 ± 0.7	*P* < .05	13.294
Talar tilt (°$\bar{\text{x}}$ ± s)				
Group A	13.7 ± 2.6	2.7 ± 1.6	*P* < .05	12.729
Group B	12.8 ± 2.9	2.3 ± 1.9	*P* < .05	16.648

Pre- and post-operatively in group A, there were 38 patients with no significant changes in the valgus angle of the foot, 5 people with reduced and 3 people with improved valgus strength. There were 42 people with no significant changes in inversion, 1 with weakened strength, and 3 with enhanced strength. No patients had a significant change in dorsal plantarflexion or dorsiflexion. After surgery, there were no significant changes in the valgus strength of the affected foot and normal foot in 30 patients. Fifteen patients had reduced valgus strength, while in 1 person it increased. There was no significant difference in normal inversion, plantarflexion, and dorsiflexion (Table 5).

**Table 5:** The changes in muscle strength of the foot.

	The affected foot pre- and postoperative		The affected foot postoperative and the normal foot	
	Valgus	Inversion	Valgus	Inversion
-∞ - -5N (Weaken)				
Group A	5	1	15	1
Group B	25	2	30	2
-5N - +5N (No change)				
Group A	38	42	30	2
Group B	15	38	12	40
+5N - +∞ (Enhance)				
Group A	3	3	1	0
Group B	2	2	0	0

## 4 Discussion

Traditional methods for surgical reconstruction of the lateral ankle ligaments include the standard open Broström-Gould technique, nonanatomic tenodesis procedures, and anatomic tendon graft. Nonanatomic reconstructions using local tendons have been reported, but some concerns arise from their invasiveness, related risks of neurovascular injuries, postoperative subtalar and tibiotalar joint stiffness, and long-term degenerative joint disease in the ankle and subtalar joint. On the other hand, anatomic repairs, more frequently performed in athletes, provide long-term stability without any impairment of range of motion.^[14]^ Anatomical reconstruction of ankle ligament refers to a surgical method for repairing injured ligament directly, and Broström-Gould method is the most representative one. This original procedure described by Broström is anatomic, minimally disruptive of local anatomy, and biomechanically advantageous, and only the ATFL could be repaired with excellent results, and recent clinical and biomechanical studies have confirmed this impression^[15,16]^ This surgical technique is easy, fast to perform, inexpensive, and provides a high rate of good to excellent outcomes in the long term,^[14]^ which merits Broström-Gould procedure might typically be carried out as a daycase procedure. However, Broström-Gould method inherit its limitations.

The study found that both the peroneus longus and peroneus brevis tendons had satisfactory results, and the ankle joints of the patients recovered mechanical stability without obvious complications. However, by comparing the effect of the long peroneal tendon with the short peroneal tendon on the valgus strength of the foot, we concluded that the long peroneal tendon is more suitable for reconstruction of the lateral collateral ligament of the ankle joint.

In this study, we treated CLAI patients with reconstruction of the lateral ligament of the ankle with the AHPLT and AHBLT. After 12 to 36 months of follow up, the preoperative and postoperative talar tilt angle, talar anterior distance, AOFAS score, VAS score, Karlsson-Peterson score, and patient subjective satisfaction were compared. The results showed that there was a significant difference between group A and group B before the operation (*P* < .05). It showed that both the peroneus longus and peroneus brevis tendons could achieve good results in the treatment of CLAI, restoring ankle joint stability, and improving patients quality of life.

There was no significant difference in the inversion strength, plantarflexion, and dorsiflexion between the AHPLT group and the AHPBT group preoperatively and at the final follow-up. It showed that AHPLT and AHBLT had little effect on inversion, plantarflexion, and dorsiflexion of the foot.

Although there was no difference in appearance between the patients in group A and group B, the valgus strength of the 2 groups was measured with a sensitive dynamometer and a statistically significant difference was observed. This indicated that the brevis longus tendon had a more important function in maintaining the valgus state. The longus is weaker, and in the treatment of lateral ankle ligament injury, the peroneus longus tendon has little effect on foot activity.

The peroneus longus tendon passes deep through the upper and lower supporting bands of the peroneal muscle. It turns forward through the back of the lateral ankle, around the sole of the foot, and runs obliquely towards the medial foot and stops at the muscles of the medial cuneiform bone and the first plantar metatarsal bone.^[17]^ Many studies have reported that AHPLT is a good donor compared with other autogenous tendons in terms of strength, safety, and morbidity of the donor site.^[5]^ In cadaveric studies, the peroneus longus tendon could bear 147.94% of the gracilis muscle, and its strength was greater than that of the gracilis muscle.^[18]^ In addition, the tension of the peroneus longus tendon and peroneus brevis tendon in fresh cadavers was measured with a unidirectional tension destruction test. The pulling force of the long peroneal tendon was 1020.4 ± 175.4 N and the short peroneal tendon was 752 ± 165.4 N. Thus, the peroneus longus tendon was 1.36 times as strong as the peroneus brevis tendon. Zhao et al used peroneal longus tendon to reconstruct the knee ligament in 92 patients. After more than 24 months of follow-up, no effects were observed in foot and ankle varus and gait. Clinical studies have shown that the AOFAS scores before and after ankle surgery were 97.4 ± 2.0 and 97.2 ± 1.6. The FADIS scores (Foot and Ankle Disability Index scores) before and after ankle surgery were 96.8 ± 2.2 and 96.9 ± 2.5, respectively. There were no signs of peroneal nerve injury, long peroneal tendon rupture, or disease.^[5]^

Peterson et al reported that the peroneus longus tendon has 2 avascular area, 1 in the lateral malleolus and the other in the cuboid bone, which are common sites of tendon rupture and tenosynovitis.^[19]^ But when we acquire the peroneus longus tendon, the tendon above the lateral malleolus is well preserved, so our technique for obtaining the tendon is less likely to cause tendon rupture or tenosynovitis than other techniques. If the patient is suspected of having peroneus tendon lesions, a preoperative MRI should be performed to prevent the risk of rupture after surgery due to the use of denatured tendons.

This study is limited by its retrospective nature. Furthermore, Owing to the short follow-up, some biomechanical complications such as residual lateral instability were not observed. Subjective assessment of the stability of the ankle joints mechanical and functional characteristics may have led to biases. The reconstruction of the lateral ligament of the ankle with autogenous tendons requires extensive and long-term follow-up in future studies.

## 5 Conclusion

The peroneus longus and peroneus brevis tendons were used to reconstruct the lateral collateral ligament of the ankle joint. The 2 methods can achieve good results and restore the stability of the ankle joint. However, the long peroneal tendon has less influence on the muscle strength of the foot, and the technique is simple, stable, and reliable, so it is worthy of being considered as a treatment. However, each patient should undergo a comprehensive, detailed, and accurate physical examination prior to surgery to provide a personalized treatment for each patient.

## Acknowledgments

The authors are grateful to K. Liu and L. Zhang of the Department of Orthopedics, and to X. Song and G. Zhang of the Department of statistics and applications for their kind assistance. We thank Peter Mittwede, MD, PhD, from Liwen Bianji, Edanz Editing China (www.liwenbianji.cn/ac), for editing the English text of a draft of this manuscript.

## Author contributions

Zongyu Yang designed the study; Fei Liu; Liang Cui, and Heda Liu inquired the EMR for variables of interest; Junshui Zuo, Sentian Li and Lin Liu searched relevant literature and analyzed and interpreted the data; Zongyu Yang wrote the manuscript and approved the final version of the manuscript.

**Conceptualization:** Lingchen Yuan.

**Data curation:** Liang Cui, Lin Liu.

**Formal analysis:** Lin Liu.

**Investigation:** Zongyu Yang, Fei Liu, Junshui Zuo, Sentian Li.

**Methodology:** Zongyu Yang, Fei Liu, Sentian Li.

**Resources:** Liang Cui, Heda Liu, Lingchen Yuan.

**Software:** Lingchen Yuan.

**Supervision:** Zongyu Yang, Heda Liu, Junshui Zuo.

**Validation:** Heda Liu.

**Visualization:** Junshui Zuo.

**Writing – review & editing:** Zongyu Yang.

## References

[1] Yeo ED Park JY Kim JH Comparison of outcomes in patients with generalized ligamentous laxity and without generalized laxity in the arthroscopic modified Brostrom operation for chronic lateral ankle instability. Foot Ankle Int 2017;107110071773033. 10.1177/1071100717730336 29034742

[2] Cienfuegos A Holgado MI Jm DDR Chronic Achilles rupture reconstructed with Achilles tendon allograft: a case report. J Foot Ankle Surg 2013;52:95–8. 22835724 10.1053/j.jfas.2012.06.006

[3] Maffulli N Ferran NA Management of acute and chronic ankle instability. J Am Acad Orthop Surg 2008;16:608–15. 18832604 10.5435/00124635-200810000-00006

[4] Sammarco GJ Idusuyi OB Reconstruction of the lateral ankle ligaments using a split peroneus brevis tendon graft. Foot Ankle Int 1999;20:97–103. 10063977 10.1177/107110079902000205

[5] Zhao J Huangfu X The biomechanical and clinical application of using the anterior half of the peroneus longus tendon as an autograft source. Am J Sports Med 2012;40:662–71. 22174343 10.1177/0363546511428782

[6] Kim HN Jeon JY Dong Q Lateral ankle ligament reconstruction using the anterior half of the peroneus longus tendon. Knee Surg Sports Traumatol Arthrosc 2015;23:1877. 24841944 10.1007/s00167-014-3072-8

[7] Sun Y Wang H Tang Y Reconstruction of the lateral ankle ligaments using the anterior half of peroneus longus tendon graft. Foot Ankle Surg 2017. 10.1016/j.fas.2017.11.001 29409185

[8] Jung HG Shin MH Park JT Anatomical reconstruction of lateral ankle ligaments using free tendon allografts and biotenodesis screws. Foot Ankle Int 2015;36:1064–71. 25921199 10.1177/1071100715584848

[9] Wang W Xu GH Allograft tendon reconstruction of the anterior talofibular ligament and calcaneofibular Ligament in the treatment of chronic ankle instability. BMC Musculoskelet Disord 2017;18:10.1186/s12891-017-1492-6 PMC5385052 28388886

[10] Kitaoka HB Alexander IJ Adelaar RS Clinical rating systems for the ankle-hindfoot, midfoot, hallux, and lesser toes. Foot Ankle Int 1994;15:349–53. 7951968 10.1177/107110079401500701

[11] Karlsson J Peterson L Evaluation of ankle joint function: the use of a scoring scale. Foot 1991;1:15–9.

[12] Jolman S Robbins J Lewis L Comparison of magnetic resonance imaging and stress radiographs in the evaluation of chronic lateral ankle instability. XXXX 2017;107110071668552. 10.1177/1071100716685526 28061547

[13] Lee SY Kwon SS Park MS Is there a relationship between bone morphology and injured ligament on imaging studies and laxity on ankle stress radiographs? Int J Sports Med 2016;37:1080–6. 27676145 10.1055/s-0042-106300

[14] Maffulli N Del Buono A Maffulli GD Isolated anterior talofibular ligament Broström repair for chronic lateral ankle instability: 9-year follow-up. Am J Sports Med 2013;41:858–64. 23388673 10.1177/0363546512474967

[15] Broström L Sprained ankles. V. Treatment and prognosis in recent ligament ruptures. Acta Chir Scand 1966;132:537–50. 5972556

[16] Broström L Sprained ankles. VI. Surgical treatment of “chronic” ligament ruptures. Acta Chir Scand 1966;132:551–65. 5339635

[17] Stockton KG Brodsky JW Peroneus longus tears associated with pathology of the os peroneum. Foot Ankle Int 2014;35:346–52. 24505044 10.1177/1071100714522026

[18] Youn H Kim YS Lee J Percutaneous lateral ligament reconstruction with allograft for chronic lateral ankle instability. Foot Ankle Int 2012;33:99–104. 22381340 10.3113/FAI.2012.0099

[19] Petersen W Bobka T Stein V Blood supply of the peroneal tendons: injection and immunohistochemical studies of cadaver tendons. Acta Orthop Scand 2000;71:168–74. 10852323 10.1080/000164700317413148

